# Chronic renoprotective effect of pulsatile perfusion machine RM3 and IGL-1 solution in a preclinical kidney transplantation model

**DOI:** 10.1186/1479-5876-10-233

**Published:** 2012-11-21

**Authors:** Raphael Thuillier, Ricardo Codas, Etienne Marchand, Hubert Lathelize, Olivier Page, Alexandre Valagier, Lionel Badet, Thierry Hauet

**Affiliations:** 1 Inserm U1082, Université de Poitiers, Faculté de Médecine et Pharmacie, Poitiers, 86021, France; 2 Service de Biochimie, CHU de Poitiers, Poitiers, F-86021, France; 3 FLIRT: Fédération pour L’étude de l’Ischémie Réperfusion en Transplantation, Poitiers, F-86000, France; 4 Service d'Urologie et chirurgie de la transplantation, Pavillon V - Hôpital Edouard Herriot, 5, place d’Arsonval, Lyon, F-69437, France; 5 CHU Trousseau, Service de chirurgie vasculaire, Fédération hospitalière Tours-Poitiers, Saint-Avertin, F-37550, France; 6 Service de Chirurgie Vasculaire, CHU de Poitiers, Poitiers, F-86021, France; 7 Faculté de Médecine Lyon-Sud, Oullins Cedex, Oullins, 69921, France; 8 University Claude Bernard Lyon 1, Villeurbanne cedex, Villeurbanne, F-69622, France; 9 Réseau CENTAURE, 63, quai Magellan, Nantes, F-44000, France; 10 Plate forme IBiSA, domaine du Magneraud, BP 52, Saint Pierre d’Amilly, Surgères, F-17700, France; 11 Inserm U1082, 2 rue de la miletrie BP577, Poitiers, 86021, France

## Abstract

**Background:**

Machine perfusion (MP) of kidney graft provides benefits against preservation injury, however decreased graft quality requires optimization of the method. We examined the chronic benefits of MP on kidney grafts and the potential improvements provided by IGL-1 solution.

**Method:**

We used an established autotransplantation pig kidney model to study the effects of MP against the deleterious effects of warm ischemia (WI: 60 minutes) followed by 22 hours of cold ischemia in MP or static cold storage (CS) followed by autotransplantation. MPS and IGL-1 solutions were compared.

**Results:**

Animal survival was higher in MPS-MP and both IGL groups. Creatinine measurement did not discriminate between the groups, however MPS-MP and both IGL groups showed decreased proteinuria. Chronic fibrosis level was equivalent between the groups. RTqPCR and immunohistofluorescent evaluation showed that MP and IGL-1 provided some protection against epithelial to mesenchymal transition and chronic lesions. IGL-1 was protective with both MP and CS, particularly against chronic inflammation, with only small differences between the groups.

**Conclusion:**

IGL-1 used in either machine or static preservation offers similar levels of protection than standard MP. The compatibility of IGL-1 with both machine perfusion and static storage could represent an advantage for clinical teams when choosing the correct solution to use for multi-organ collection. The path towards improving machine perfusion, and organ quality, may involve the optimization of the solution and the correct use of colloids.

## Background

Nowadays, kidney transplantation is the best treatment for patients with end-stage renal disease, with long-term benefits on patient survival and quality of life [1]. However, this therapy is now a victim of its success, with an increasing shortage of organs: in 2010 in the USA, 15,429 kidney transplants were performed for 87,903 patients on the waiting list (OPTN, 2010). This leads to donor demographic changes as transplantation centers now accept more extended criteria donors (ECD). These donors are defined as presenting co-morbidity factors such as age, death from cardiovascular causes, cardiovascular risk factors or a reduction in glomerular filtration [2]. These conditions increase the sensitivity of the organ to preservation conditions [3]. Furthermore, there is renewed interest in deceased after cardiac death donors (DCD) [4], also displaying an increased rate of delayed graft function (DGF) and a higher incidence of primary non function [5]. The increased proportion of these types of donors has lead to re-thinking of proper organ preservation methods as in order to fully use these sources an important effort must be made to improve organ quality through better preservation.

Machine perfusion (MP) is based on a controlled circulation of a solution within the organ for the duration of the preservation. MP improves the quality of kidneys in the clinic [6] and is particularly efficient in decreasing both primary non function and DGF in ECD [7] as well as DGF in DCD transplantation [8]. These benefits are also fuelling debate about the cost effectiveness of the method and the subgroups of patients on which to use it [9]. However, despite its significant efficiency in reducing DGF and increasing three year survival [10], a high incidence of failure remains, highlighting the need for improvements to be made on this method in order to bring it to its full potential.

In a previous study, we measured the benefits of MP versus static cold storage (CS) using the Waters RM3 system in a preclinical model of DCD kidney transplantation in pigs [11,12]. In the same study, we analyzed the possible benefits of using the IGL1 (Institute George Lopez 1) solution in the system instead of the recommended MPS (Machine Perfusion Solution). Indeed IGL-1 is a fourth generation preservation solution containing polyethylene glycols (PEG), which offer several advantages in preservation solutions: they are non toxic, neutral, water soluble, and their high affinity for water molecules along their chain creates layers of ‘structured water’ when they are adsorbed to the cell surface, offering an ‘exclusion volume’ preventing cells and proteins fixations, as in the case of the immunological synapse [13], offering the possibility of ‘immunocamouflage’ [14]. Protection from the immune system is also provided by the effect of PEG binding on cell surface potential gradient, affecting charge-charge and non-covalent binding/adhesion involved in antigen–antibody recognition. We determined in this short term study that there was a clear protection from MP when compared to CS, independently of the solution, and that there was a slight advantage from using MPS over IGL1 in MP, as kidneys preserved with the later showed some increase in the level of tissue injury recorded on an anatomopathological scale.

Herein we propose the results of the aforementioned study with a longer follow up (3 months), with the aim to provide more discriminating data between the groups. Indeed, 3 months after transplant is the time of choice for protocol biopsies in patients, allowing for estimation of the development of chronic lesions such as interstitial fibrosis and tubular atrophy. In our pig model, we previously showed that 3 months was also a valuable time to evaluate the chronic consequences of preservation, particularly regarding chronic injuries.

We performed these experiments in a pig model of kidney transplantation, reproducing conditions of DCD, which present the advantage of using the same machines as the clinic [11]. In this setting, we subject the kidney to 60 min of warm ischemia prior to 22 hours of hypothermic pulsatile perfusion in RM3, a model reflective of the uncontrolled DCD (Maastricht classes I and II). It provides pre clinically relevant information comparing the effects of MP with the commonly used static cold storage solutions. The model allows the direct assessment of IRI and renal and tubular cell function without the complication of immunogenic factors or recipient characteristic. In addition, porcine kidneys are of similar size and weight than adult human kidney and are one of the two biomedical animals (with large primates) presenting a multilobular and multipapillary kidney and an elaborate system of interlobular and segmental arteries to supply the numerous kidney lobes, it is therefore well adapted for the modeling of kidney transplantation [15]. The present study uses an isograft model, devoid of the influence of both brain death and immunosuppressants. This route was chosen because we felt that immunosuppressors, with their own set of deleterious side effects, would bias our results. Furthermore, machine perfusion has been developed to optimize graft preservation, hence address ischemia reperfusion injury, leading us to run this first series of tests on a non-brain dead animal. It is also important to note that our model is designed to follow the setting of classes I and II of the Maastricht criteria, but does not fit it exactly. Indeed, this criteria includes no more than 30 min arrest before starting the CPR procedure, which is then continued during the transport to the hospital (generally with a machine); then as failure to resuscitate is pronounced there is a 5 min no touch period. All these steps should not exceed 150 min before femoral vessels canulation. The donor is then either cold perfused or an extracorporeal circuit is put in place, giving enough time to secure consent from the family and collect the organs, which are then machine perfused. Our current model does not include all these steps, however we trust that 60 min WI reproduces as closely as possible the conditions of DCD. Lastly, although our model does not include brain death and thus cannot be used to draw direct conclusions in regards to ECD organ transplantation, the high degree of injury encountered by the organ suggest that any intervention proven beneficial in this model is likely to improve ECD organ quality and thus would warrant a dedicated investigation with an appropriate model.

## Methods

### Animal model and surgical procedure

Left kidneys from 28 large white pigs aged 4 weeks and weighting 40 ± 4 kg were collected after vascular clamping of the renal vessels for 60 min (warm ischemia, WI) preceded by administration of 150 U/kg heparin 10 min before clamping the left/or right renal artery. Then the organ was removed and immediately cold-flushed with 500-600 mL of preservation solution at a constant pressure of 75 mmHg (Belzer MPS or IGL-1 placed at 1 m of height). There was no difference in time or volume of flush out between the groups. Kidneys were preserved during 22 hours at 4°C. Four groups were studied: **MPS-CS:** static incubation with Belzer MPS (n = 7); **MPS-MP:** renal perfusion with Belzer MPS using the RM3 Waters Medical Systems pulsatile machine (n = 7); **IGL-CS:** with IGL-1 solution in static conservation (n = 7); **IGL-MP:** renal perfusion with IGL-1 (n = 7). Afterwards, the kidney was re-implanted and the contralateral was removed. Heparin (5000U) was injected prior to clamping pre-anastomosis. The heterotopic autotransplantation of the left kidney using end-to-side aorta and vena cava anastomosis was performed via the mid-line incision, as previously reported [16]. In all cases, re-warming ischemia during implantation until anastomosis was 30 ± 5 min. A control group of 3 animals was performed to assess the normal values of creatinine in pigs with a single nephrectomy. All animal experiments were conducted according to the guidelines of the French Ministry of Agriculture for the use and care of laboratories animals.

### Renal function evaluation

Pigs were placed in metabolic cages for 24-h urine collections. Blood and urinary samples were collected. Plasma creatinine and proteinuria were measured with a Modular bioanalyser (Roche Diagnostics, France).

### Morphological study

Corticomedullar kidney samples were collected at 3 months. A standard procedure was used to estimate the level of tubulointerstitial fibrosis using Picro-Sirius staining [17] on 5–10 fields (100X). For immunohistofluoresence, anti-Vimentin antibody (Cell Marque, Rocklin, CA, USA) was used with secondary antibody coupled with Cy3 (Invitrogen). Quantitative evaluation was performed *in silico* on 35 high powered fields (5 per animal, 200X).

### Real Time quantitative PCR (RTqPCR)

We used Trizol for RNA extraction (Fisher Scientific, France). Genomic DNA was removed using DNA-free kit (Applied Biosystems, USA) and first-strand reverse transcription (Applied) was performed. Real Time PCR assays were performed on an ABI Prism 7300 (Applied). Porcine primers (Additional file 1: Table S1) were designed using OligoPerfect™ (Invitrogen, USA). Expression level was obtained with the 2^(−ΔΔCt)^ Method. Controls are age-matched kidneys which have not beet subjected to ischemia reperfusion.

### Statistical methods

Results are shown as mean±SEM. For the statistical analysis among groups, we used NCSS software (NCSS LLC, USA) and one-way ANOVA analysis. In case the ANOVA revealed statistical differences, Tukey-Kramer post-hoc test for multiple comparisons in case of normality (Skewness, Kurtosis and Omnibus tests) and equality of variance (Modified-Levene Equal-Variance Test) and Kruskal-Wallis Multiple-Comparison Z-Value Test (Dunn's Test) in case these parameters were not met. For the multivariate analysis of variance, the Wilks' Lambda, Hotelling-Lawley Trace, Pillai's Trace and Roy's Largest Root tests were used to evaluate the hypothesis that the solution and/or the machine had no effect on the variable, and in case the hypothesis was rejected the analysis of variance for each factor and for their interaction was conducted. Statistical significance was accepted for P < .05.

## Results

### Early function recovery

Compared to MPS-CS, the decrease in serum creatinine levels post-transplantation was faster in the MPS-MP, IGL-CS and IGL-MP groups (Table 1), particularly evidenced by the levels reached at day 14. There was no statistically significant difference between these three groups.

**Table 1:** Kidney function recovery after reperfusion

**Groups**	***Serum Creatinine (μmol/L)***			
	**D-1**	**D5**	**D7**	**D14**
**MPS CS**	89.3 ± 4.6	1475.1 ± 72.4	1531.6 ± 407.4	1254.4 ± 655.7
**MPS MP**	92.1 ± 4.8	1225.7 ± 184.7	1225.7 ± 184.7 *	179.4 ± 20.2 *
**IGL CS**	81.0 ± 4.4	1135.3 ± 161.8	1135.3 ± 161.8 *	168.4 ± 19.5 *
**IGL MP**	82.0 ± 5.8	1389.3 ± 158.3	1389.3 ± 158.3 *	213.6 ± 19.1 *

Values are mean ± SEM. Statistics: *: p < 0.05 versus MPS-CS; ‡: p < 0.05 to IGL CS; ¶: p < 0.05 to MPS MP.

### 3month survival and function

**Figure 1:**
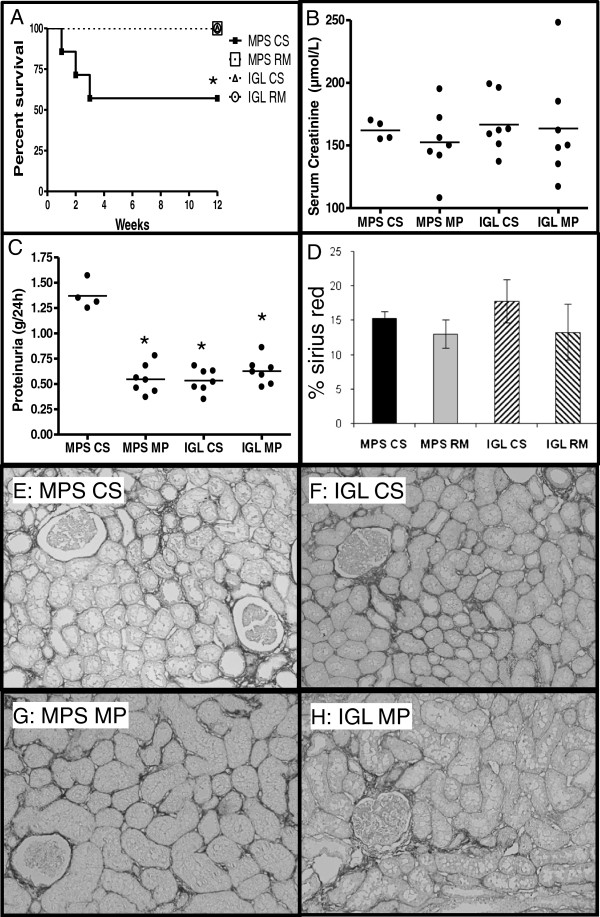
**Functional and histological phenotype at 3 months.A**-**B**: Animal Survival and graft function. A: Kaplan-Meier representation of animal survival.** B**: Serum creatinine and **C**: proteinuria. **D**-**H**: Interstitial Fibrosis and Tubular Atrophy at 3 months. **D**: Quantification of staining in each group **E**-**H**: Representative red Sirius staining for all 4 conditions. Shown are mean ± SEM. Statistics: *: P < .05 versus MPS-CS; ‡: P < .05 to IGL CS; ¶: P < .05 to MPS MP.

At the end of the follow up, only 57% of animals in the MPS-CS group survived while all animals of the other groups were able to reach the end of the follow up (Figure 1A, P < .05). Animal loss during follow up was due to renal failure, 3 cases of animal loss due to surgical errors (1 anastomosis failure, 2 bowel misplacement) were excluded from the analysis. No sign of vascular thrombosis was observed. Renal function of surviving animals showed no differences between the groups in terms of serum creatinine (Figure 1, B). There was a higher level of proteinuria in the MPS-CS group compared to the other three groups (Figure 1, C, P < .05).

### Chronic fibrosis development

Analysis of interstitial fibrosis intensity revealed similar levels in all groups (around 15%), typical for fibrosis in its earlier stages (Figure 1 D-H).

### Activation of injury mechanisms

**Figure 2:**
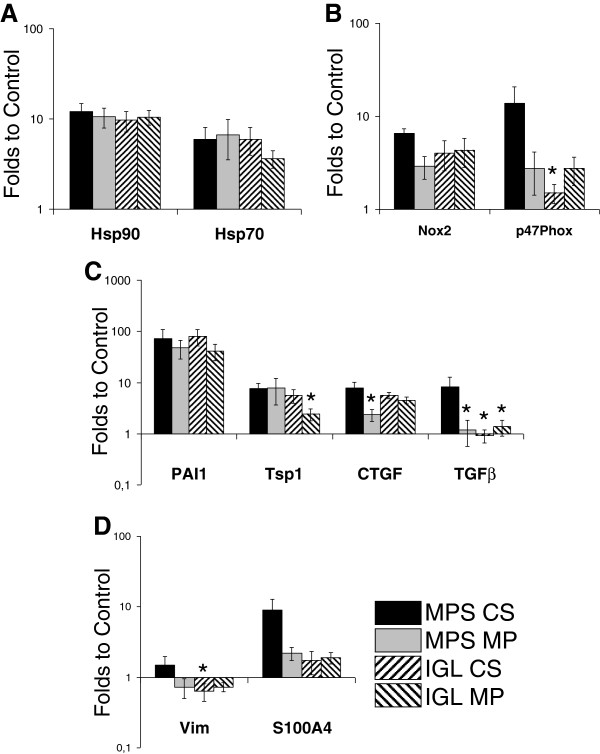
**RTqPCR of chronic stress and fibrosis factors at 3 month.** Quantification of gene expression was computed as stated in Methods. Shown are mean ± SEM. Statistics: *: P < .05 versus MPS-CS; ‡: P < .05 to IGL CS; ¶: P < .05 to MPS MP.

We performed RTqPCR on markers of chronic kidney lesion pathways and determined that grafts from all groups demonstrated heightened levels of stress, as shown by heat shock proteins (Hsp) 70 and 90 expression (Figure 2, A). Oxidative stress was also present, with equivalent expression of NADPH oxidase subunit Nox 2 in all groups, while p47phox subunit showed higher expression in the MPS-CS group compared to the other three groups (Figure 2, B).

### Activation of fibrosis development mechanisms

We measure the expression of pro-fibrosis markers by RTqPCR (Figure 2 C) and determined a similar level of overexpression for PAI1 in all groups. Thrombospondin 1 (Tsp1) was also overexpressed in all groups although slightly reduced in IGL-MP. Connective tissue growth factor (CTFG) overexpression was detected in all group, with some reduction in MPS-MP kidneys. Interestingly, expression of transforming growth factor β (TGFβ) was only detected in MPS-CS grafts.

Investigating mediators of epithelial to mesenchymal transition (Figure 2, D), we measured a trend towards overexpression of Vimentin in MPS-CS kidneys compared to the other groups, seemingly confirmed by S100A4 measurements.

### Epithelial to mesenchymal transition (EMT)

**Figure 3:**
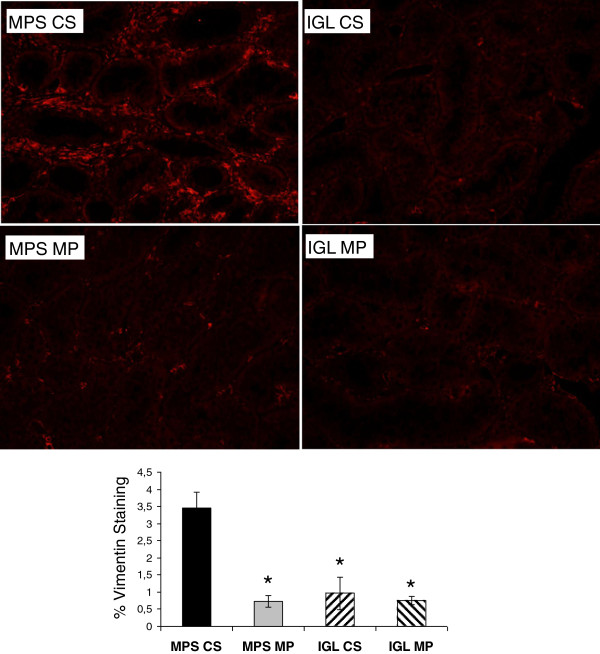
**Epithelial to Mesenchymal transition at 3 months.** Top: Representative Vimentin staining for all 4 conditions. Bottom: Quantification of staining in each group. Shown are mean ± SEM. Statistics: *: P < .05 versus MPS-CS; ‡: P < .05 to IGL CS; ¶: P < .05 to MPS MP.

We performed immunohistofluorescent staining for Vimentin and determined that in concordance with RTqPCR data, staining intensity was high in the MPS-CS Group (Figure 3) while it remained low in all three other groups (P < .05).

### Chronic inflammation markers

**Figure 4:**
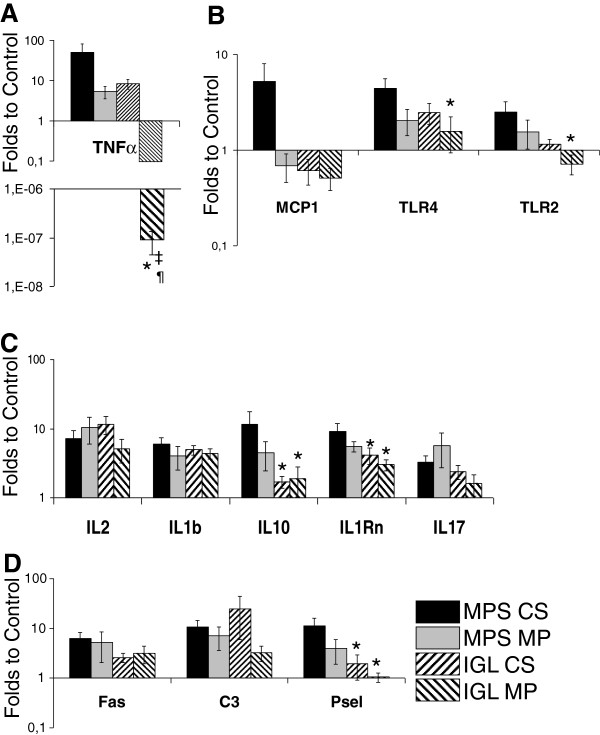
**RTqPCR of inflammation factors at 3 month.** Quantification of gene expression was computed as stated in Methods. Shown are mean ± SEM. Statistics: *: P < .05 versus MPS-CS; ‡: P < .05 to IGL CS; ¶: P < .05 to MPS MP.

We further defined the inflammatory situation by RTqPCR. Expression of TNFα was high in MPS-CS group while it appeared lowered in both MPS-MP and IGL-CS groups. IGL-MP group showed highly decreased expression for this marker, suggesting a likely negative regulation of inflammation at the time of analysis (Figure 4, A). This trend was also observed in other innate immunity markers, as MCP1 appears overexpressed in the MPS-CS group compared to the other two (Figure 4, B). TLR4 and TLR2 expression analysis revealed a similar trend, with significantly lower expression in IGL-MP compared to MPS-CS (Figure 4, B). IGL groups also showed significantly lowered expression of adaptative markers IL10 and IL1Rn compared to MPS-CS (Figure 4, C), and diminished expression of endothelial activation marker P selectin (Figure 4, D).

### Multivariate analysis of variance

In order to measure the influence of the preservation solution (MPS or IGL1) and the mode of preservation (MP or CS) on organ quality, both their individual contribution and their interaction, we performed a multivariate analysis of variance on proteinuria and vimentin staining at 3 months (Table 2).

**Table 2:** Multivariate analysis of variance results

***Factor***	***Vimentin Staining at 3 months***				
	***Sum of Squares***	***Mean Square***	***F-Ratio***	***Probability level***	***Power***
**A: Solution**	6,801158	6,801158	10,39	0,006132	0,849834
**B: Mode of Preservation**	9,683869	9,683869	14,79	0,001782	0,946426
**AB: Interaction**	7,065302	7,065302	10,79	0,005421	0,862812
**S: Residual variance**	9,16562	0,6546872			
	***Protéinuria at 3 months***				
***Factor***	***Sum of Squares***	***Mean Square***	***F-Ratio***	***Probability level***	***Power***
**A: Solution**	0,844812	0,844812	48,59	0,000001	0,999998
**B: Mode of Preservation**	0,788403	0,788403	45,35	0,000001	0,999996
**AB: Interaction**	1,239591	1,239591	71,30	0,000000	1,000000
**S: Residual variance**	0,3650857	1,738503E-02			

The results show that each factor plays an important individual role for these parameters, as demonstrated by the high F ratio and low p value. Interestingly, the influence of the interaction between solution and mode of preservation was also high, indicating that the improvement brought by the combination of factors is more than the simple addition of their individual contribution.

## Discussion

In the present study, we confirmed previous findings demonstrating the benefits of MP on chronic kidney graft outcome [11]. Considering the importance of the solution bias when measuring the impact of machine preservation [18] and the fact that we previously demonstrated the superiority of MP over CS, all other parameters being equal [12,19], we did not include in the present study a CS group using standard static preservation solution (for instance University of Wisconsin) as we felt a repetition of these experiments would represent an unethical use of animals, in addition, an non-randomized prospective multi-center study showed that in clinical practice IGL-1 solution has the same effectiveness as UW solution in cold storage [20]. However, in previous studies using the same DCD model with static University of Wisconsin preservation, we found serum creatinine levels of approximately 400 μmol/L at 3 months [21], which when compared to the data of the present suggests superiority of MP versus CS when these modes are used in clinic-like conditions. Nevertheless, use of MPS for both CS and MP permitted the measurement of the benefits of the RM3 without interference from solution components, and indeed when using MPS, recommended solution for the RM3 machine, we show that MP increases graft survival and preserves chronic function parameters such as proteinuria compared to static storage. However, other typical parameters such as serum creatinine and fibrosis were not changed by the machine. In depth analysis of the phenotypes within the kidney graft by RTqPCR showed decreased levels in the expression of markers typical of chronic lesion such as Notch 4, which has been shown to inhibit angiogenesis [22], thus likely impeding the proper reparation mechanisms within the graft. We also detected lower p47phox expression in MPS-MP group, a subunit of NADPH oxidase. NADPH oxidase is a protein complex located in the plasma membrane as well as in the membrane of phagosome, which variation in expression have been linked to the level of oxidative stress in the tissue and to chronic injury [23]. Thus, MP appears beneficial in regards to chronic stress on the parenchyma.

Another important mechanism of chronic kidney graft lesion is epithelial to mesenchymal transition, during which epithelial cells differentiate and alter their phenotype towards that of a mesenchymal cell, thus going from a polarized, anchored and non dividing cell to an unpolarized, mobile and fast proliferating cell. This regenerative process is believed to be deregulated and promote fibrosis in the context of chronic graft rejection [24]. We investigated this phenomenon by first using RTqPCR to observe expression of Vimentin, typical marker of EMT, and CTGF, an important downstream effector of TGF-β involved in fibrosis [25] and EMT [26]. In our study, MPS-MP grafts had decreased expression of these markers compared to MPS-CS graft, a result which was then confirmed by immunohistofluorescence for Vimentin.

Hence, although interstitial fibrosis measurement using standard method (Sirius Red Staining) did not reveal a difference between these groups, mechanistic analysis showed that this process was slowed down in the MP group compared to the CS group. These results suggest that uncovering MP benefits may be difficult using standard methods of evaluation. We previously came to a similar conclusion during the evaluation of the Lifeport machine [19], when the use of KPS, of identical composition than MPS, made comparison between MP and CS more difficult as there was only slight differences in survival and typical biochemical evaluation. There also, proteinuria was one of the most discriminating markers.

In this previous study as well as the present one, the bias of the perfusion solution was very apparent. In both studies, multivariate analyses revealed both an individual effect of the machine and the solution on outcome markers, but more importantly showed a significant effect of the combination of factors. Our results herein show that the evidence for MP benefits is difficult to find when using IGL1 in CS and MP.

We previously reported little differences between IGL1 and MPS in MP when follow up was limited to 30 days, with a slight advantage for the use of MPS in terms of histological injury. Herein, longer follow up highlighted differences between both IGL groups and MPS-CS as evidenced by several parameters such as survival and proteinuria, as well as lesional markers detected by RTqPCR. Both IGL groups displayed reduced expression of TGFβ and Vimentin, which was confirmed by immunostaining, suggesting a better level of protection obtained through the use of the PEG-containing solution.

The use of PEG involves the concept of immunocamouflage [14], we thus investigated the immune response within the grafts. Interestingly, classical evaluation of immune cell invasion did not reveal differences between the groups (data not shown), however RTqPCR evaluation of immune markers determine some effects of IGL compared to MPS. IGL preserved grafts presented reduced levels of IL-10 and IL-1Rn, typical of Th2 phenotype. As Th2 immunity is linked to the development of fibrosis [27], the protection provided by IGL appears to be directed against fibrotic pathways.

Further investigation of innate immunity by RTqPCR analysis revealed that expression of TNFα, TLRs and Pselectin was the lowest in IGL grafts. TNF-α is one of the earliest cytokine produced in response to stresses, particularly by resident dendritic cells [28]. TLRs are well described effectors of ischemia reperfusion injury [29] and TLR4 as recently been shown to have a central role in kidney graft injury, both on the short and long term [30]. Pselectin is a marker of endothelial activation. Hence, the use of IGL appears to lower the immunogenicity of the graft, confirming our previous findings in an allotransplantation model [31].

The benefits from combining IGL and MP were more difficult to determine. Indeed, the only statistical difference between the IGL-CS and IGL-MP groups was in the recorded level of TNFα expression, which was significantly lower than controls, suggesting a negative regulation taking place within the kidney at the time of analysis. More data points would be necessary to describe in further detail the dynamics of immune regulation in this context. However, the difficulty in assessing the benefits of the combined use of IGL1 and MPS is concordant with retrospective and prospective studies published in the literature which have shown the benefit of MP [32] but did not clearly demonstrate the independent effect of MP with different preservation solutions. However, IGL-MP group results were more consistently reaching statistically significant difference to MPS-CS compared to the other groups. Thus, combining MP and IGL increases the protective potential of each technique to better preserve the graft against chronic adverse outcomes.

The difficulty in discriminating between groups with classical approached such as histology and biochemistry underlines the need for tools providing in depth analysis of the tissue phenotypes. Herein we demonstrate that although two tissues show similar levels of fibrosis, one group is much further along on the path towards fibrosis development according to the transcripts expressed by the cells. These results highlight the need for further research on in depth biomarkers, particularly using highly reproducible models to identify specific reporters of injury linked to variations in a single parameter.

In conclusion, this study demonstrates that the evaluation of machine perfusion is highly dependant on the solution used. Multivariate analysis of variance confirmed that the perfusion solution was significantly associated with the result, and moreover that combination of solution and machine could have additive effects. IGL-1 used in either machine or static preservation offers similar levels of protection than standard MP. Importantly, our data is in contradiction with a previous report from our team with a shorter follow up time. This highlight the needs for proper follow up durations in preclinical studies, particularly in research areas where chronic outcome is of crucial importance. The compatibility of IGL-1 with both machine perfusion and static storage could represent an advantage for clinical teams when choosing the correct solution to use for multi-organ collection. The path towards improving machine perfusion, and organ quality, may thus involve the optimization of the solution and the correct use of colloids.

## Competing interests

The authors declare that they have no competing interests**.**

## Authors' contribution

RT performed RT-PCR and immunihistochemical experiments, analysed data and wrote the paper. RC performed animal experiments and gathered data. EM performed histophathological experiments and analysed data. HL performed biochemistry measurement. OP performed animal experiments and gathered data. AV performed biochemistry and RT-PCR experiments and analysed data. LB designed the study and managed data gathering/analysis. TH designed the study, managed project and wrote the paper. All authors read and approved the manuscript.

## Supplementary Material

Additional file 1 **Table 1.** Primer sequences for RT-PCR analysis in Pig Kidneys. Click here for file
