# Supplementary material for: Chronic renoprotective effect of pulsatile perfusion machine RM3 and IGL-1 solution in a preclinical kidney transplantation model
Source: J Transl Med. 2012 Nov 21;10:233. doi: 10.1186/1479-5876-10-233 (PMC3543357; doi:10.1186/1479-5876-10-233)
Supplement: Additional file 1 — Table 1. Primer sequences for RT-PCR analysis in Pig Kidneys. [file 1479-5876-10-233-S1.doc]

**SUPPLEMENTARY TABLES**

Table 1 : Primer sequences for RT-PCR analysis in Pig Kidneys.

| Gene | Forward | Reverse |
| --- | --- | --- |
| 18S | AGCCTGCGGCTTAATTTGAC | AACCAGACAAATCGCTCCAC |
| C3 | GCTGACCCCTATGAGGTTGT | GATCTCCACCTGCTCATTGC |
| CTGF | GGCGGCTTACCGACTG | TGGAACAGGCACTCCACTC |
| Fas | CCTGGCAAACGGAAACAT | CTTCTAGGCCGTGTTCTCCA |
| HIF1α | TGGCAGCAATGACACAGAAAC | GAGGCAGGCAATGGAGACAT |
| Hsp70 | TTTTCTGCCTCCACAAACG | ATCTAATGCAAAGAATACAGTCCAG |
| Hsp90 | CCGGTGCCGATATCTCGAT | GCGACCAGGTACGCAGAGTAG |
| IL-10 | GCTCCAAGAGAGGGGTGTC | CGTCATGTAGGCTTCTATGTAGTTG |
| IL-17 | TCACTGCTGCTTCTGCTGAG | GTCCTCAGTTTTTGGGCATC |
| IL-1b | GAAGTGCTGCACCCAAAAC | TCTGCCTGATGCTCTTGTTC |
| IL-1Rn | TTGGACGCAGTTAACATCACA | GGAGCGGATGAAGGTGAAG |
| IL-2 | AGCAGGCTACAGAATTGAAACA | GAGTCAGAGTTTTTGCTTTGACC |
| MCP1 | TCTCCAGTCACCTGCTGCTAT | TGCTTCTTTAGGACACTTGCTG |
| Notch4 | CAGTCCAAGCAAAAATGCCAG | GTGTGGAGATGCAGCCGC |
| Nox2 | GTGCACCATGATGAGGAGAA | AGTTAGGCCGTCCGTACAAG |
| P47phox | CATCATCCTGCAGACGTACC | TCGTTCTTCTCCACGACATC |
| PAI1 | TTGAGGAGAAGGGCATGG | CATCGGCCGTGCTG |
| P-selectin | TGATCTAATGAACCAGAAGGGAGT | CTCGCGAAGTATTCCATGAGT |
| S100A4 | GGAAAAGGACGGATGAAGC | GAAGACGCAGTACTCCTGGAA |
| TGFβ | CGAGCCAGAGGCGGACTA | TATCATAGATTTGGTTGCCGCTTT |
| TLR2 | GGGCTCTGTGCCACCACTT | TGGAGCCAGGCCCACAT |
| TLR4 | GCTTTTTGTGGGCTGCAAA | GGAGTAGATAACAAAGGCGTCATAGG |
| TNFα | TCAGATCATCGTCTCAAACCTC | CCAGCTGGTTGTCTTTCAGC |
| Tsp-1 | CCTAATCATCAAACTGTTGACCA | GAGTCCACACTGATGCAAGC |
| Vimentin | AGGTGGACCAGCTCACCAA | ATGATGTCCTCGGCCAGATT |
